# Impact of Phytase Supplementation on Meat Quality of Heat-Stressed Broilers

**DOI:** 10.3390/ani13122043

**Published:** 2023-06-20

**Authors:** Clay J. Maynard, Craig W. Maynard, Garrett J. Mullenix, Alison Ramser, Elizabeth S. Greene, Mike R. Bedford, Sami Dridi

**Affiliations:** 1 Department of Poultry Science, University of Arkansas, Fayetteville, AR 72701, USA; 2 Bell & Evans, Fredericksburg, PA 17026, USA; 3 AB Vista, Marlborough SN8 4AN, UK; mike.bedford@abvista.com

**Keywords:** broilers, growth, muscle myopathy, meat quality, heat stress, phytase, gene expression

## Abstract

**Simple Summary:**

The adverse effects of heat stress on poultry production sustainability are well known, however, there is still a paucity of information regarding its effect on meat quality. Here, we report the effect of heat stress and supplementation of exogenous phytase on growth performances, muscle myopathy incidence, and meat quality as well as its underlying molecular mechanisms in broilers.

**Abstract:**

Heat stress (HS) is one of the most challenging stressors to poultry production sustainability. The adverse effects of HS range from feed intake and growth depression to alteration of meat quality and safety. As phytase supplementation is known to improve nutrient utilization and consequently growth, we undertook the present study to evaluate the effects of dietary phytase on growth and meat quality in heat-stressed broilers. A total of 720 day-old hatch Cobb 500 chicks were assigned to 24 pens within controlled environmental chambers and fed three diets: Negative Control (NC), Positive Control (PC), and NC diet supplemented with 2000 phytase units (FTU)/kg) of quantum blue (QB). On day 29, birds were exposed to two environmental conditions: thermoneutral (TN, 25 °C) or cyclic heat stress (HS, 35 °C, 8 h/d from 9 a.m. to 5 p.m.) in a 3 × 2 factorial design. Feed intake (FI), water consumption (WI), body weight (BW), and mortality were recorded. On day 42, birds were processed, carcass parts were weighed, and meat quality was assessed. Breast tissues were collected for determining the expression of target genes by real-time quantitative PCR using the 2^−ΔΔCt^ method. HS significantly increased core body temperature, reduced feed intake and BW, increased water intake (WI), elevated blood parameters (pH, SO_2_, and iCa), and decreased blood pCO_2_. HS reduced the incidence of woody breast (WB) and white striping (WS), significantly decreased drip loss, and increased both 4- and 24-h postmortem pH. Instrumental L* and b* values were reduced (*p* < 0.05) by the environmental temperature at both 4- and 24-h postmortem. QB supplementation reduced birds’ core body temperature induced by HS and improved the FCR and water conversion ratio (WCR) by 1- and 0.5-point, respectively, compared to PC under HS. QB increased blood SO_2_ and reduced the severity of WB and WS under TN conditions, but it increased it under an HS environment. The abovementioned effects were probably mediated through the modulation of monocarboxylate transporter 1, heat shock protein 70, mitogen-activated protein kinase, and/or glutathione peroxidase 1 gene expression, however, further mechanistic studies are warranted. In summary, QB supplementation improved growth performance and reduced muscle myopathy incidence under TN conditions. Under HS conditions, however, QB improved growth performance but increased the incidence of muscle myopathies. Therefore, further QB titration studies are needed.

## 1. Introduction

Growing concerns about the effects of global warming on animal agriculture have become apparent over the last decade. The United States poultry industry produced a total of 59.5 billion live pounds of chicken in 2021 [1]. In addition, the average weight of market-ready broilers increased by 0.05 pounds while maintaining feed conversion ratio (FCR) [1]. However, the problem associated with this net gain in productivity was at the expense of an increase of half a percent in mortality from the previous year [1]. Scientists speculate that growing concerns about global warming have aided in this increase in mortality noted in the poultry industry [2,3]. Not only do terminal effects become present with increasing temperature, but heat stress has been noted to depress feed intake, increase feed conversion ratio, reduce processing yield, and negatively affect meat quality in poultry [4,5,6,7]. Furthermore, reduced functionality in meat products is apparent when heat stress has been observed in broiler rearing [8]. Thus, the poultry industry is investigating ways to reduce the negative effects that heat stress causes on broiler production and final meat quality as global temperatures continue to rise.

Broiler efficiency has undeniably continued to improve as feed conversion and genetic selection have advanced. Improvements have primarily been accredited to genetic selection (>70%), but nutritional strategies have continued to aid in success [9,10]. Quantum Blue (QB; AB Vista) is a commercial phytase supplement proven to improve FCR in the modern broiler [11]. The limiting effects on the performance of phytate are practically and efficiently reduced with the inclusion of phytase in the diet. Performance advancements then improve financial gain to the industry and provide a greater means for return. The advantageous effects of QB in live performance have also recently gained interest as a potential aid in meat quality. Although not directly linked, our lab previously defined a reduction in woody breast (WB) severity in broilers supplemented with QB at 2000 FTU/kg [12]. However, that experiment lacked assessments of other meat quality factors, providing a need to further assess meat quality in QB diets. In addition, the literature lacks a definition regarding how phytase supplementation can impact broiler meat quality.

The negative impacts of environmental condition imbalance partnered with the advantageous performance noted with phytase inclusion provide promising results for improvements in broilers. In addition, the limited knowledge of the mode of action of phytase inclusion on meat quality needs to be further defined. Therefore, this experiment was conducted to assess the effects of chronic cyclic HS and in feed-phytase inclusion on meat quality characteristics in broilers.

## 2. Materials and Methods

### 2.1. Animal Husbandry, Experimental Design, and Diet Treatments

A total of 720 day-old male broiler chicks (Cobb 500 by-products, Cobb-Vantress, Inc., Siloam Spring, AR, USA) with an average BW of 39 ± 0.12 g were allotted randomly to 12 controlled environmental chambers in the Poultry Environmental Research Laboratory at the University of Arkansas (2 pens/chamber, 30 birds/pen, density of 0.096 m^2^ per bird). Each pen was covered with clean pine wood shavings and equipped with separate feeders and jug waterers. Birds were given ad libitum access to clean water and feed for the duration of the study (42 d). The ambient temperature was gradually decreased from 32 °C for days 1 to 3, 31 °C for days 4 to 6, 29 °C for days 7 to 10, 27 °C for days 11 to 14, and 25 °C thereafter. A relative humidity of ~30–40% was maintained and the lighting program was 24 h light for the first three days, reduced to 23 h light:1 h dark from day 4 to 7, and then reduced further to 18 h light:6 h dark thereafter. The environmental temperature and humidity were continuously recorded in each pen using HOBO pro V2 data loggers (ONSET, Bourne, MA, USA).

From day 1, birds were assigned randomly to 1 of 3 diet treatments comprising a basal starter (d1–18), grower (d19–28), and finisher (d29–42) diet (nutrient adequate positive control, PC, Table 1), a diet with a reduction of available phosphorus, calcium and sodium by 0.15, 0.16 or 0.03%, respectively, as a negative control (NC), and an NC diet supplemented with phytase (Quantum Blue, QB, 2000 FTU/kg, AB Vista, Marlborough, UK). At day 29, birds were exposed to 2 environmental conditions: thermoneutral (25 °C) or chronic cyclic heat stress (35 °C for 8 h/day from 8:30 a.m. to 4:30 p.m.) to mimic summer season in Arkansas, USA, which resulted in a total of 6 treatments in 3 × 2 factorial arrangements (3 diets × 2 environmental condition factorial designs, 4 pens/treatment, 120 birds/treatment). Feed and water intake were measured on a daily basis from d1–42. One day before the onset of HS, two chickens per pen were randomly selected and a Thermochron temperature logger (iButton, Embedded Data Systems, Lawrenceburg, KY, USA) was placed in the crop via oral gavage for continuous monitoring of core body temperature as previously described [13,14].

### 2.2. Blood Parameters

Blood chemistry, gas, and electrolytes were measured using a portable analyzer (i-STAT Alinity, Abbott Laboratories, Abbott Park, IL, USA; cartridge Cg8+) as we previously described (Baxter, et al., 2020 [14]). Briefly, blood was collected (2 birds/pen, 8 birds/group) at d42 and introduced into the cartridge using a syringe, and the cartridge is then inserted into the analyzer, and operator and animal identification were entered into the system.

### 2.3. Processing

Upon completion of a 10-h feed withdrawal period, birds were transported to the University of Arkansas Pilot Processing Plant. Upon arrival, broilers were weighed on the back dock, hung on an inline shackle system, and processed as previously described [15]. Briefly, broilers were electrically stunned (11 V, and 11 mA for 11 s), exsanguinated, scalded in hot water (53.8 °C, 2 min), and then defeathered. Prior to mechanical evisceration, necks and hocks were manually removed from each bird. Carcasses were then subjected to a two-stage chilling system consisting of a 0.25 h prechill at 12 °C before being placed in immersion chilling tanks held at 0 °C for 2.5 h with manual agitation. At 3 h postmortem, carcasses were deboned to determine *Pectoralis major* (breast) and *P. minor* (tender), wing, and whole leg weights. *Pectoralis major* muscles were then utilized for further analysis with the following parameters: color, pH, and drip loss.

### 2.4. Color

At 4- and 24-h postmortem, intact left fillets had their color recorded with a handheld Minolta colorimeter and data were configured using SpectraMagic NX software (Minolta CM-400, Konica Minolta Sensing Americas Inc., Ramsey, NJ, USA), set with a 2-degree observer, decreasing surface reflectance, and illuminant parameters of D65. Before measuring, the colorimeter was calibrated to CIE specifications using a white calibration tile agreeing with the procedure provided for poultry by the American Meat Science Association (AMSA) [16]. Intact fillets were positioned dorsal side up on white storage trays to record measurements on the left fillet. Three separate L*, a*, and b* values were measured for each fillet in the cranial, medial, and caudal locations which were then subsequently averaged.

### 2.5. pH

Immediately after the color was recorded, the pH of each left fillet was measured. Left halves were evaluated at 4- and 24-h postmortem for pH using a spear-tip pH probe with automatic temperature compensation (Model 205, Testo instruments, West Chester, PA, USA). Samples were collected by inserting the pH probe near the wing joint area of each fillet and allowing it to equilibrate until a reading was maintained for three seconds.

### 2.6. Drip Loss

After pH and color readings were recorded at 4-h postmortem, fillets were placed on white plastic storage trays, wrapped in plastic overlay liners, and placed in a walk-in cooler held at 4 °C until 24-h postmortem. At 24-h postmortem, breast fillets were removed from the cooler and reweighed for determination of drip loss. Drip loss was calculated as a percent by weight in relation to deboned weight.

### 2.7. RNA Extraction, Reverse Transcription, and Quantitative Real-Time PCR

Total RNAs were extracted from chicken left-beast muscle tissues using Trizol reagent (Life Technologies, Grand Island, NY, USA) according to the manufacturer’s recommendations. After DNAse treatment and purification, total RNA concentrations were determined for each sample by a Take 3 Micro-Volume Plate using a Synergy HT multi-mode micro plate reader (BioTek, Winooski, VT, USA), and RNA integrity and quality were assessed by both OD260/OD280 nm absorption ratio (1.8–2) and by using 1% agarose gel electrophoresis. For cDNA synthesis, total RNA (1 µg) was reverse transcribed using qScript cDNA SuperMix (Quanta Biosciences, Gaithersburg, MD, USA) in a 20 µL total reaction. The reverse transcription reaction was performed at 42 °C for 30 min followed by incubation at 85 °C for 5 min. Real-time quantitative PCR (Applied Biosystems 7500 Real-Time PCR system, Applied Biosystems, Waltham, MA, USA) was performed using 5 µL of 10× diluted cDNA, 0.5 µM of each forward and reverse specific primer, and PowerUp SYBR Green Master Mix (ThermoFisher Scientific, Rockford, IL, USA) in a total 20 µL reaction as previously described [17,18,19]. Oligonucleotide primers specific for chicken superoxide dismutase 1 and 2 (SOD1/2), glutathione peroxidase 1 and 3 (GPX1/3), heat shock protein 60, 70, and 90 (HSP60/70/90), Mitogen-activated protein kinase 1, 3, 8, and 14 (MAPK1/3/8/14), collagen type 1 alpha 1 and alpha 2 chains (Col1A1, Col1A2), and r18S as a housekeeping gene were previously reported [10,20,21]. Primer sequences specific for the other target chicken genes were: monocarboxylate transporter 1 (MCT1, Genebank accession# NM_001006323): Forward 5′-GCATCTTTGGGAGTTCTGTTGAT-3′ and reverse 5′-CCTATCGTGCTCCAACAAACC-3′; MCT2 (Genebank accession# NM_001199604) forward 5′-TCTGGGTTTGGCATTCAACTT-3′ and reverse 5′-CGCTTCTTATAGAAATACTTGCCAATC-3′; MCT3 (Genebank accession# NM_205140) forward 5′-GGGTCCGCCCTCATGTG -3′ and reverse 5′-TGTCGAGCCATTGAAGAGCAT-3′; glycogen synthase (GYS2, Genebank accession # NM_001406729) forward 5′-CATTGACAAGGAAGCAGGAGAGA-3′ and reverse 5′-GTGTACAGATGCCCGTTCCA-3′; glucose 6 phosphatase catalytic 2 (G6PC2, Genebank accession # XM_003641648) forward 5′-GCCTCGAACAGTTTCCCATAAC-3′ and reverse 5′-CCCATGGCATGTCCAGATG-3′; glucose 6 phosphatase catalytic 3 (G6PC3, Genebank accession # XM_015273297) forward 5′-CCGACCCCAAATGCATCTT-3′ and reverse 5′-CTTGCGGTCGAGGAAATAGG-3′; glycogen branching enzyme (GBE1, Genebank accession # XM_015298401) forward 5′-AAGAAAATGGAGTATGGGAAATGG-3′ and reverse 5′-TGAGGCACAGGAGAAAAACCA-3′ and glycogen debranching enzyme (AGL, Genebank accession # XM-040677682) forward 5′-TTCTAGCGTTTGGTGGGACTCT-3′ and reverse 5′-CCCTGGCCAAGCAGGTT-3′. Relative expressions of target genes were determined by the 2^−ΔΔCt^ method [22]. Samples extracted from birds maintained under thermoneutral conditions and fed the NC diet were used as a calibrator.

### 2.8. Statistics

Data were analyzed by Two-way ANOVA and Tukey’s HSD multiple comparison test using the Mixed Model platform of JMP Pro 16.2 (SAS Institute, Cary, NC, USA) for the growth and meat quality data, and Graph Pad Prism version 8.00 for Windows (Graph Pad Software, La Jolla, CA, USA) for the blood electrolytes, gas, glucose, hematocrit, and hemoglobin as well as the muscle gene expression. For the growth data, pen served as the experimental unit and pen averages were calculated and analyzed for each quality characteristic. For the gene expression, however, the bird was used as the experimental unit. Statistical significance was set at *p* ≤ 0.05. When the interaction between environment and diet is not significant, the main effect of diet or environment was analyzed independently by One-Way ANOVA and Tukey or by Student T test as appropriate.

## 3. Results

### 3.1. Core Body Temperature, Growth Performances, and Blood Parameters

**Figure 1:**
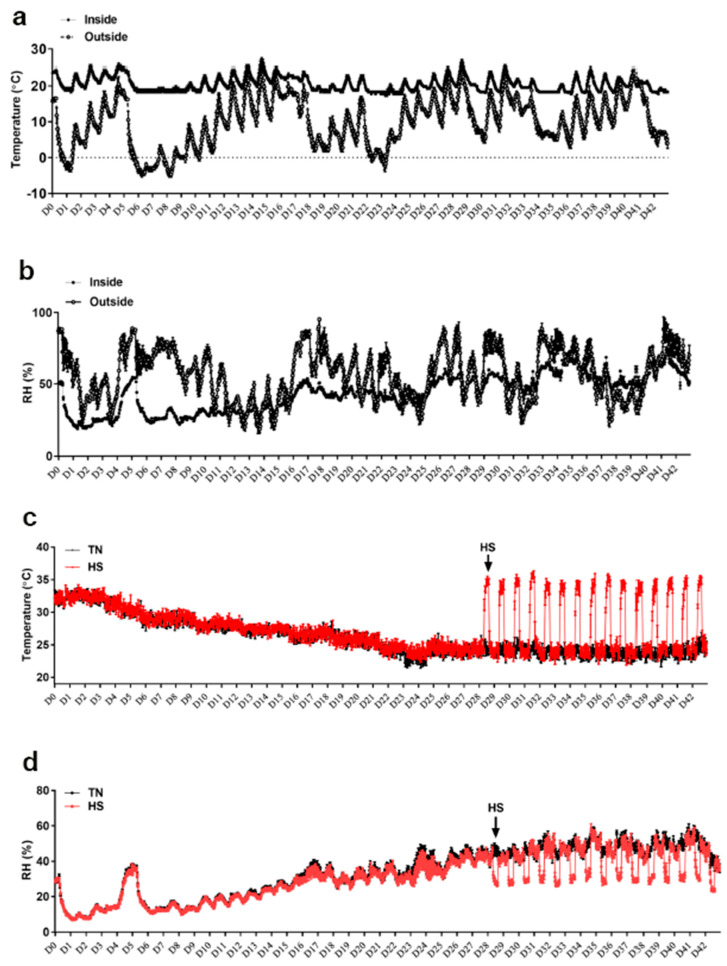
Temperature and relative humidity (RH) fluctuations in the barn (**a**,**b**) and in the environmental chambers (**c**,**d**), during the cyclic heat stress experiment. Birds raised under recommended conditions from d0–28. From d29–42, birds either raised at thermoneutral temperature (25 °C) or exposed to chronic cyclic heat stress (35 °C, 8 h/day, from 9:30 a.m. to 5:30 p.m.). Data are presented as mean ± SEM (*n* = 1/chamber or 8/group).

The experiment was conducted from 2 February to 29 March 2022. As depicted in Figure 1a, the average outside environmental temperature varied between −5 °C to 20 °C, however, the temperature inside the barn was maintained between 19 °C to 25 °C. The outside RH was approximately 25% to 90%, however the RH inside the barn was about 25% to 50% (Figure 1b). Despite the abovementioned variations, the cyclic temperature inside the environmental chambers was successfully maintained as planned (35 °C from 8:30 a.m. to 4:30 p.m., and 25 °C during the rest of the day, Figure 1c). The RH inside the chambers averaged between 15% to 40% from d1–29, and then cyclically between 15% during the HS period to ~40% during the rest of the day from d29–42 (Figure 1d).

**Figure 2:**
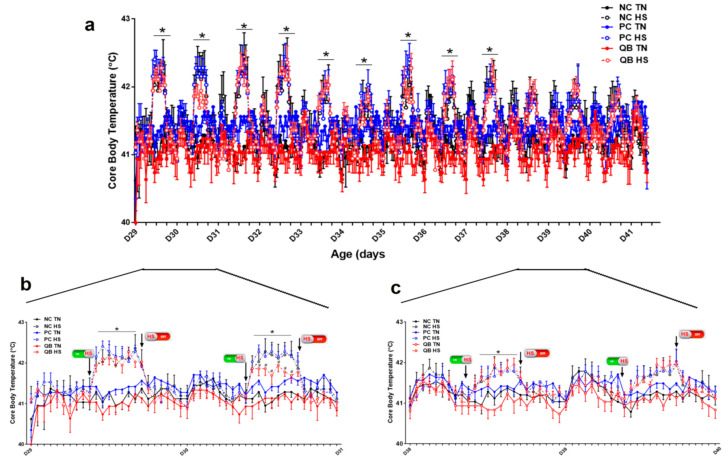
Core body temperature fluctuations during chronic cyclic heat stress experiment (HS). (**a**) represents the whole HS period from d29–41, (**b**,**c**) represent a detailed daily variation from d29–d3 and d38–40, respectively. Birds were fed three diets: NC, PC, or QB and exposed to two environmental conditions: TN, 25 °C or HS (35 °C, 8 h/d) in a 3 × 2 factorial design as described in materials and methods. Data are presented as mean ± SEM (*n* = 2 birds/pen or 8 birds/group). * indicates significant difference at *p* < 0.05. HS, heat stress condition; NC, negative control diet; PC, positive control diet; QB, quantum blue-supplemented diet; TN, thermoneutral condition.

The cyclic 10 °C increase in ambient temperature significantly elevated the birds’ core body temperature by approximately 0.5–1 °C from d29 to d38, and the amplitude was reduced from d38 onward (Figure 2a). This elevation occurred within 2 h of the daily rise in environmental temperature and returned to TN levels as the ambient temperature returned to 25 °C (Figure 2a). Daily plots showed that QB supplementation reduced the birds’ core body temperature under TN conditions from d29 to d41, and under HS only from d29 to d38 (Figure 2b,c).

**Figure 3:**
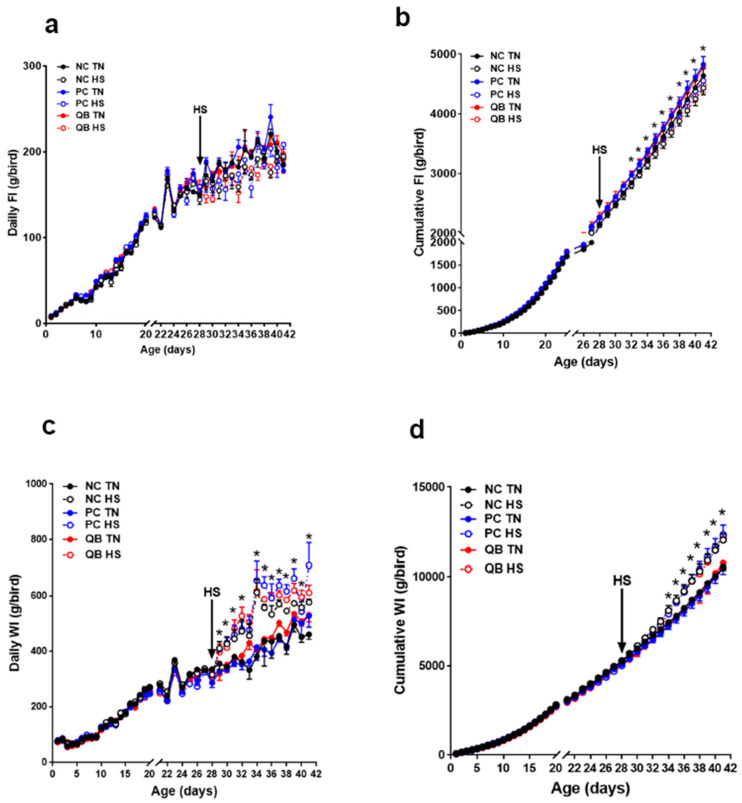
Effects of QB supplementation on feed intake and water consumption of broilers maintained at TN conditions or exposed to chronic cyclic HS. (**a**) daily FI, (**b**) cumulative FI, (**c**) daily WI, and (**d**) cumulative WI. Data are presented as mean ± SEM (*n* = 4 pens/group, 120 birds/group). * indicates a significant difference (*p* < 0.05) compared to TN birds. FI, feed intake; HS, heat stress condition; NC, negative control diet; PC, positive control diet; QB, quantum blue-supplemented diet; TN, thermoneutral condition; WI, water intake.

**Figure 4:**
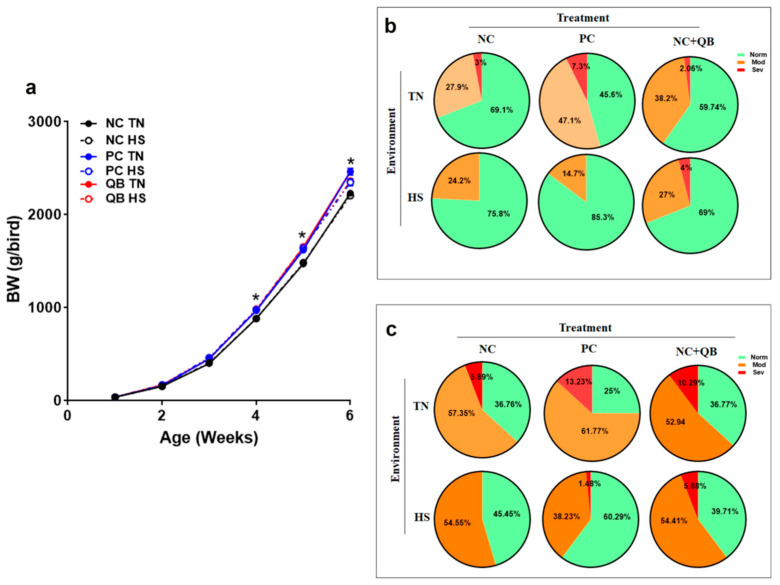
Effects of QB supplementation on body weight (**a**) and muscle myopathy incidences (**b**,**c**) in broilers maintained at TN conditions or exposed to chronic cyclic HS. At day 42 and after the slaughter process, breast filets were macroscopically scored and classified to WB and WS categories to normal (NORM, score 0), moderate (MOD, score 0.5–1.5), and severe (SEV, score 2–3). Data for BW are mean ± SEM (*n* = 120 birds/group), and data for myopathy incidence are presented as %. * indicates a significant difference (*p* < 0.05) compared to NC-TN birds.

As expected, HS exposure significantly decreased daily feed intake (FI) from d29 and cumulative FI from d30 (Figure 3a,b) until the end of the experiment (d42), which in turn resulted in a significant decrease in body weight (BW) by approximately 80 g (Figure 4a and Table 2) and this decrease was more pronounced in PC compared to the NC and NC + QB groups. During HS exposure (d29–42), FCR was significantly decreased, which resulted in an overall decline in FCR at the conclusion of the experiment, although the difference was not statistically discernable (Table 2). Both PC- and QB-supplemented diets increased BW under both environmental conditions, however, the latter group ate numerically less feed and performed 49- and 10-points better FCR compared to the former under TN and HS conditions, respectively (Figure 3a,b and Table 2).

HS significantly increased daily and cumulative WI (Figure 3c,d), as well as the WCR at the conclusion of the experiment (*p* < 0.0001, Table 2). Compared to the PC group, QB supplementation increased WCR by 24 points under the TN condition, but it reduced it by 5 points under the HS environment (Table 2).

HS increased blood pH (*p* = 0.03), SO_2_ (*p* = 0.04), and iCa (*p* < 0.01), but it decreased that of pCO_2_ (*p* = 0.02) and glucose concentrations (*p* < 0.01) compared to the TN condition (Table 3). A significant effect of diet was discerned for SO_2_ with a clear increase in the QB group compared to the PC group under both environmental conditions (Table 3). QB supplementation also increased blood SO_2_ (*p* = 0.02), pH (*p* = 0.06), and pO_2_ (*p* = 0.06), but it reduced pCO_2_ (*p* = 0.06) compared to PC birds (Table 3).

### 3.2. Meat Quality and Muscle Myopathy Incidence

For meat quality and as summarized in Table 4, an interaction was only present between diet and environmental temperature for both 4- and 24-h postmortem pH values (*p* < 0.05). No other interaction was observed during this experiment (*p* > 0.05). HS significantly reduced drip loss in breast fillets 24-h postmortem. Breast fillet pH was significantly increased by HS in NC + QB birds at 4 h postmortem, but significantly decreased in all heat-stressed birds at 24-h postmortem (Table 4). Instrumental L* and b* values were reduced (*p* < 0.05) by the environmental temperature at both 4- and 24-h postmortem. A main effect of diet was only present for breast b* values. For both 4- and 24-h b* values, the NC diet expressed b* values greater than both the QB and PC diets (*p* = 0.014 and 0.013, respectively). There were no main effects observed (*p* > 0.05) for dietary treatment or environmental temperature, or an interaction, on a* values at the conclusion of the experiment.

The total incidence of WB was reduced by HS from 30.9% to 24.2% in NC birds, 54.4% to 14.7% in PC, and from 40.26% to 31% in the QB group (Figure 4b). When categorized, severe WB was totally absent in heat-stressed NC and PC birds, but it increased from 2.06% to 4% in QB-fed birds (Figure 4b). Under TN conditions, QB supplementation reduced the incidence of severe WB compared to NC and PC diets (Figure 4b). Similar to WB, HS decreased the total incidence of white striping (WS) from 63.24% to 54.55% in the NC group, from 75% to 39.71% in the PC group, and from 63.23% to 60.29% in the QB group (Figure 4c). HS also reduced the incidence of severe WS from 5.89% to 0% in NC, from 13.23% to 1.48% in PC, and from 10.29% to 5.88% in the QB group (Figure 4c). QB supplementation reduced the incidence of severe WS compared to the PC group under TN, but it increased it under HS conditions (Figure 4c).

### 3.3. Expression of Muscle Molecular Markers

**Figure 5:**
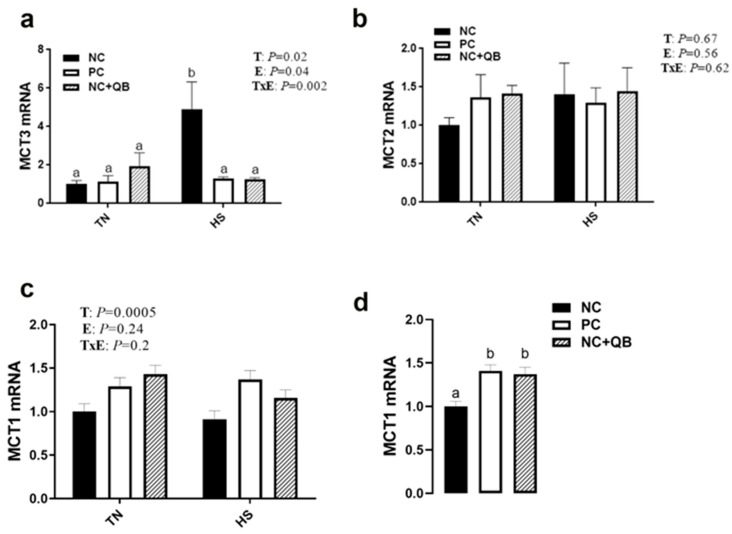
Effects of QB supplementation on breast muscle MCT gene expressions in broilers maintained at TN conditions or exposed to chronic cyclic HS. Relative expression of the MCT3 (**a**), MCT2 (**b**), and MCT1 (**c**,**d**) genes was determined by qPCR and analyzed by the 2^−ΔΔCt^ method [22] using the NC-TN group as a calibrator. Data are presented as mean ± SEM (*n* = 8 birds/group). Different letters indicate significant differences at *p* < 0.05. E, environment; HS, heat stress; MCT, monocarboxylate transporter; NC, negative control diet; PC, positive control diet; QB, quantum blue; T, treatment; TxE, treatment by environment interaction; TN, thermoneutral.

**Figure 6:**
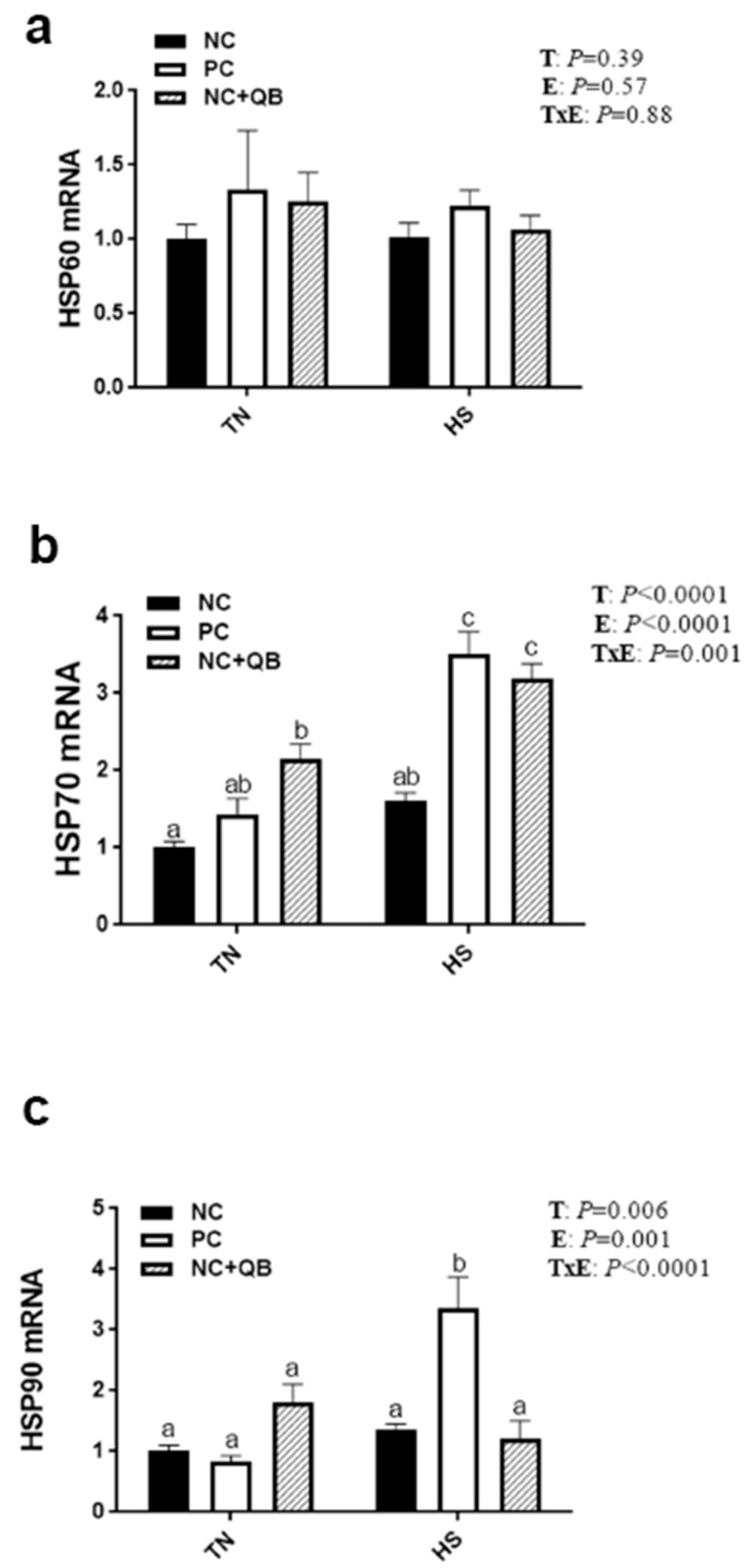
Effects of QB supplementation on breast muscle HSP gene expressions in broilers maintained at TN conditions or exposed to chronic cyclic HS. Relative expression of the HSP60 (**a**), HSP70 (**b**), and HSP90 (**c**) genes was determined by qPCR and analyzed by the 2^−ΔΔCt^ method [22] using the NC-TN group as a calibrator. Data are presented as mean ± SEM (*n* = 8 birds/group). Different letters indicate significant differences at *p* < 0.05. E, environment; HS, heat stress; HSP, heat shock protein; NC, negative control diet; PC, positive control diet; QB, quantum blue; T, treatment; TxE, treatment by environment interaction; TN, thermoneutral.

**Figure 7:**
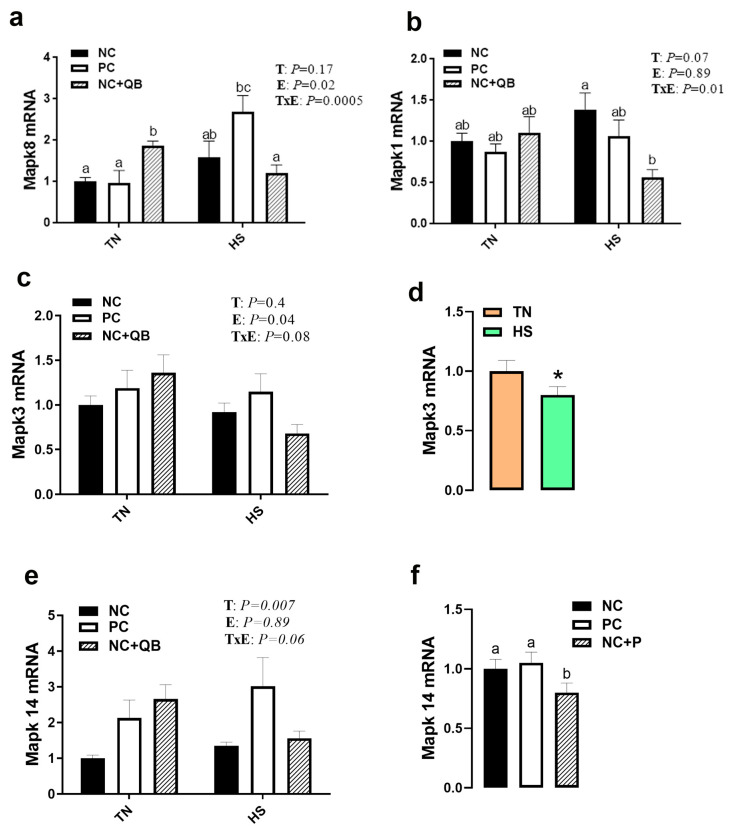
Effects of QB supplementation on breast muscle Mapk gene expressions in broilers maintained at TN conditions or exposed to chronic cyclic HS. Relative expression of the Mapk8 (**a**), Mapk1 (**b**), Mapk3 (**c**,**d**), and Mapk14 (**e**,**f**) genes was determined by qPCR and analyzed by the 2^−ΔΔCt^ method [22] using the NC-TN group as a calibrator. Data are presented as mean ± SEM (*n* = 8 birds/group). * and different letters indicate significant differences at *p* < 0.05. When the TxE interaction was not significant, the main effect (environment or diet) was analyzed independently (**d**,**f**). E, environment; HS, heat stress; Mapk, mitogen-activated protein kinase; NC, negative control diet; PC, positive control diet; QB, quantum blue; T, treatment; TxE, treatment by environment interaction; TN, thermoneutral.

**Figure 8:**
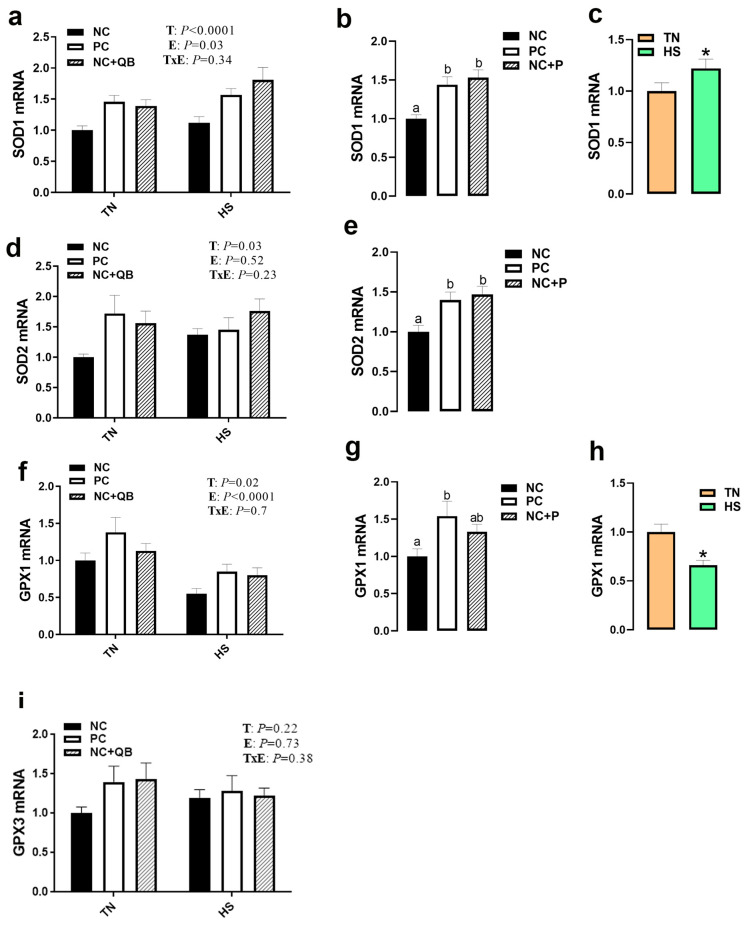
Effects of QB supplementation on the expression of breast muscle antioxidant system-associated genes in broilers maintained at TN conditions or exposed to chronic cyclic HS. Relative expression of the SOD1 (**a**–**c**), SOD2 (**d**,**e**), GPX1 (**f**–**h**), and GPX3 (**i**) genes was determined by qPCR and analyzed by the 2^−ΔΔCt^ method [22] using the NC-TN group as a calibrator. Data are presented as mean ± SEM (*n* = 8 birds/group). * and different letters indicate significant differences at *p* < 0.05. When the TxE interaction was not significant, the main effect (environment or diet) was analyzed independently (**b**,**c**,**e**,**g**,**h**). E, environment; GPX, glutathione peroxidase; HS, heat stress; NC, negative control diet; PC, positive control diet; QB, quantum blue; SOD, superoxide dismutase; T, treatment; TxE, treatment by environment interaction; TN, thermoneutral.

**Figure 9:**
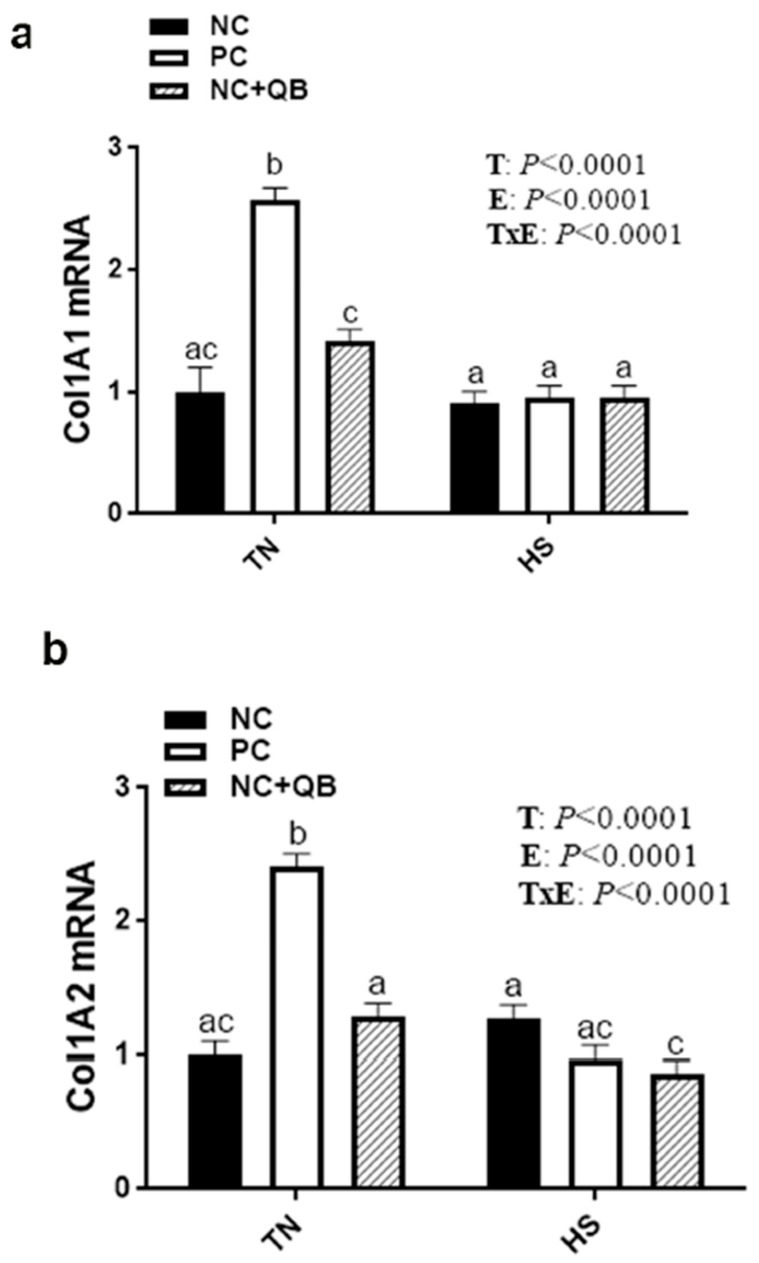
Effects of QB supplementation on breast muscle collagen gene expression in broilers maintained at TN conditions or exposed to chronic cyclic HS. Relative expression of the Col1A1 (**a**) and Col1A2 (**b**) genes was determined by qPCR and analyzed by the 2^−ΔΔCt^ method [22] using the NC-TN group as a calibrator. Data are presented as mean ± SEM (*n* = 8 birds/group). Different letters indicate significant differences at *p* < 0.05. Col1A1, collagen type 1 alpha 1 chain; Col1A2, collagen type 1 alpha 2 chain; E, environment; HS, heat stress; NC, negative control diet; PC, positive control diet; QB, quantum blue; T, treatment; TxE, treatment by environment interaction; TN, thermoneutral.

**Figure 10:**
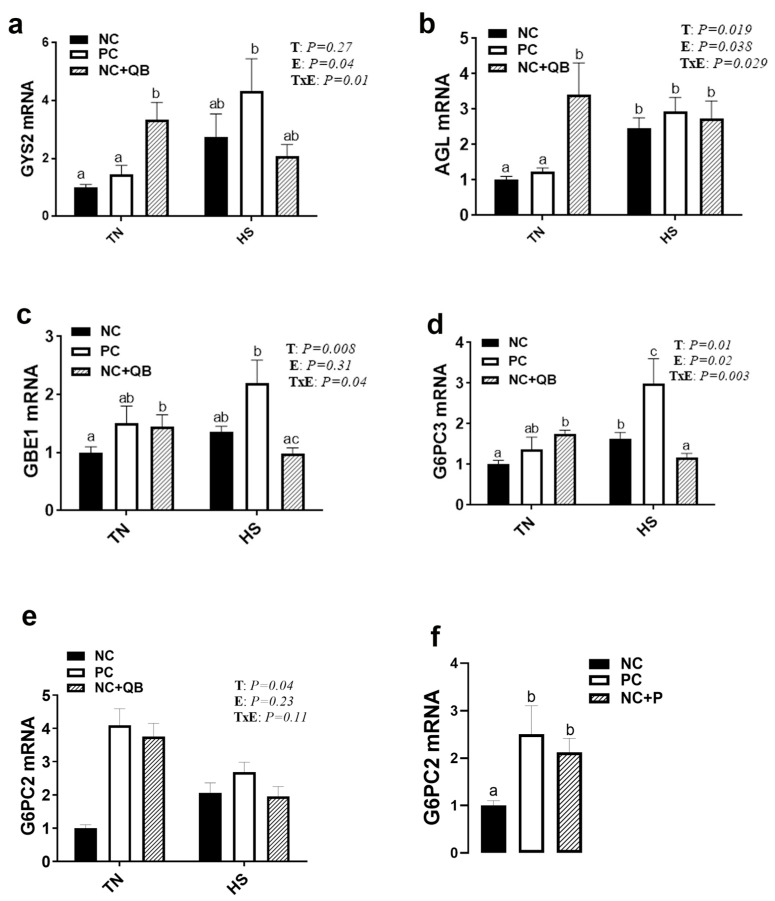
Effects of QB supplementation on the expression of breast muscle glycogenolytic/gluconeogenic pathways in broilers maintained at TN conditions or exposed to chronic cyclic HS. Relative expression of the GYS2 (**a**), AGL (**b**), GBE1 (**c**), G6PC3 (**d**), and G6PC2 (**e**,**f**) genes was determined by qPCR and analyzed by the 2^−ΔΔCt^ method [22] using the NC-TN group as a calibrator. Data are presented as mean ± SEM (*n* = 8 birds/group). Different letters indicate significant differences at *p* < 0.05. When the TxE interaction was not significant, the main effect of diet was analyzed independently. AGL, glycogen debranching enzyme; E, environment; G6PC, glucose 6 phosphatase catalytic; GBE1, glycogen branching enzyme; GYS, glycogen synthase; HS, heat stress; NC, negative control diet; PC, positive control diet; QB, quantum blue; T, treatment; TxE, treatment by environment interaction; TN, thermoneutral.

HS exposure altered the expression of muscle MCT3, HSP70, HSP90, Mapk3 (ERK1), Mapk8 (JNK), SOD1, GPX1, Col1A1, Col1A2, and glycogenesis/glycogenolysis-related genes. mRNA abundances of MCT3 were significantly induced by HS in only the NC group (Figure 5c), which resulted in a significant diet x environment interaction (*p* = 0.002). HS upregulated the expression of muscle HSP70 in the PC and QB groups (Figure 6b), HSP90 in only the PC group (Figure 6c), Mapk8 in the PC group (Figure 7a), GYS2 in the PC group (Figure 10a), AGL in both NC and PC birds (Figure 10b), and G6PC3 in the NC and PC groups (Figure 10d). A significant interaction between diet and environment was discerned for the abovementioned genes. On the other hand, HS exposure significantly downregulated the expression of muscle Mapk3, Mapk8 in the QB group (Figure 7a,c), GPX1 (Figure 8f,h), Col1A1 and Col1A2 in the PC and Qb groups (Figure 9a,b), and GBE1 and G6PC3 in QB birds (Figure 10c,d). The expression of MCT1, MCT2, and GPX3 was not affected by HS (Figure 5b,c and Figure 8i).

Compared to NC, both PC- and QB-supplemented diets significantly upregulated breast MCT1, SOD1, and SOD2 gene expression (Figure 5c,d and Figure 8b,e). Compared to the PC diet, QB supplementation significantly down regulated the expression of HSP90 (Figure 6c), Mapk8 (Figure 7a), Mapk14 (Figure 7e,f), GBE1 (Figure 10c), and G6PC3 (Figure 10d) in heat-stressed birds, and that of Col1A1 and Col1A2 in TN birds (Figure 9a,b). However, QB supplementation increased the breast muscle expression of Mapk8 (Figure 7a), GYS2 (Figure 10a), and AGL (Figure 10b) under TN conditions.

## 4. Discussion

HS is one of the most challenging stressors and a stumbling block to poultry production sustainability because of its multifaceted detrimental effects. The strong adverse effects of HS on growth performance, welfare, health, and mortality, as well as its heavy economic impacts, are well documented [23,24,25,26,27,28]. However, scientific reports related to the effect of HS on poultry meat quality are very limited, but increasingly emerging [29,30,31]. The present study aimed, therefore, to further define basic mechanisms associated with the effects of HS on broiler meat quality, and to determine whether phytase supplementation ameliorates HS productivity losses at both performance and quality levels.

In a continuum of previous studies, including our own, HS increased core body temperature and depressed feed intake as expected [32,33,34], with the latter being the most prominent known effect of HS that might contribute to the decline of heat gain from digestion and metabolism [35]. Parallel to these changes and at the conclusion of the experiment (d42), HS reduced FCR in all studied groups. Interestingly, QB-fed birds had numerically higher BW than the other groups under both environmental conditions, and less BW loss and better FCR compared to the PC group under HS conditions. These results are anticipated because exogenous phytase fed at high doses has been reported to induce a rapid and complete breakdown of phytate in diets, reducing the anti-nutritional effects, and thereby eliciting greater nutrient utilization and consequently better growth [36,37]. It is also possible that the beneficial effects of QB observed in our experimental HS conditions are mediated through the downregulation of Mapks (Mapk1 or ERK2, Mapk8 or JNK1, and Mapk14 or P38), which are involved in stress response and cell death [38,39,40,41]. Moreover, numerous reports have shown that these kinases can be localized in a different cellular compartment such as endosomes where they play key roles in nutrient intake/digestion [42], or mitochondria which has a pivotal role in energy metabolism, redox biochemistry, and survival/death decision [43,44].

Of particular interest, HS significantly increased overall WCR compared to the TN condition at the conclusion of the trial (d42). Intriguingly and independently of the environmental conditions, QB supplementation significantly increased WCR compared to other groups, particularly from d29–42, which might be associated with electrolyte profile and extracellular acid-base balance [45,46] which need further in-depth investigations.

HS reduced the incidence of both muscle myopathies, WB and WS, which probably have different etiologies but are often concomitant. WS first appeared in 2009 [47] and is visually characterized by white striation (fat) parallel to muscle fibers on the breast, tender, and thigh [48]. WB, however, emerged around 2014 [49], and is denoted by a tougher consistency to raw breast filets [50]. Histologically, both myopathies are characterized by myodegeneration, necrosis, fibrosis, and lipidosis [51], and appeared in varying degrees, often together on the same filet. They are emerging at a large scale and gaining the attention of poultry scientists and poultry meat producers globally. The question that remains to be answered is what mechanisms mediate the reducing effects of HS on muscle WB and WS incidence? The first logical thought is the reduction of BW, as these myopathies have been observed in modern fast-growing and heavy birds [52]. However, as the incidence is sporadic with varying degrees of severity, this could not be the only answer. Second, it is possible that HS affects WB incidence via hypoxia, but this is counterintuitive and unlikely in our experimental conditions because hypoxia has been reported by our group and others to play a key causative role in WB myopathy progression and development [53], and HS is well known to induce hypoxia-like status in internal organs as hot birds divert blood to the skin to dissipate heat [54]. Thus, one would expect that HS could increase WB incidence, which was not the case here. Similarly, HS has been reported to alter lipid metabolism in chickens by inducing visceral, subcutaneous, and intramuscular fat deposition [55,56], and consequently one would expect that this may increase the WS incidence, however, the opposite data were observed. It is also plausible that HS affects these incidences via modulation (activation, proliferation, and/or differentiation) of satellite cells [57,58], which warrants further functional and mechanistic studies.

When assessing meat quality, the interaction of environmental temperature and diet on 4- and 24-h pH appears to be apparent more as a tendency in difference between the QB diets in each environmental condition, rather than amongst all three diets within each environmental condition. This interesting interaction may be related to phytase activity, yet the authors do not fully understand the effects of the interaction. There also appears to be a lack of resources available in the literature that pertain to these combined effects which further exacerbates a possible explanation for this interaction. Thus, the need to further investigate this relationship is merited. Heat-stressed broilers expressed a lower drip loss than their TN counterparts and previous studies have reported that HS has a detrimental effect on broiler meat quality and can produce meat that is pale, soft, and exudative (PSE-like) in nature [5,59]. The PSE-like phenomenon, however, is known to produce larger quantities of drip loss when compared to normal meat [60]. That trend was reversed in the current experiment and has proven to be consistent and aligns with recent studies [61], where the authors suggested that this might be due to excessive dehydration and cellular osmosis being readily apparent in HS broilers producing lower quantities of free water loss in deboned meat.

Coincide this aspect of drip loss with the final ultimate pH, and the explanation for the results in HS broilers becomes more complex. Primarily resultant of rigor mortis development, pH is affected by the accumulation of lactic acid as a by-product of anaerobic glycolysis [5]. Following harvest, birds are depleted of their oxygen supply, forcing the production of energy to an anaerobic cycle and the accumulation of lactic acid to begin [62]. In HS broilers, glycogen stores are utilized during excessive panting, transport, and/or during holding times at the processing plant [63], and this is supported here by the alteration of glycogen metabolism-associated genes, namely glycogen synthase and the branching enzyme (AGL) as well as glucose-6-phosphatase catalytic subunits that play key roles in the glycogenolytic/gluconeogenic pathways [64]. This accelerated use of stored energy reserve continues upon exsanguination, resulting in rapid pH decline within the first few hours postmortem [8]. This acidified muscle, at the cost of severe lactic acid accumulation, leads in turn to PSE-like meat. The overall divergence of pH values at 24-h postmortem in the current study aligns with numerous previously conducted experiments regarding PSE-like meat [5,63,65]. Rapid pH decline is typically observed in PSE-like meat within the early postmortem phase, but the prolonged effect of cyclic heat stress in the current experiment produced final resting pH values (24-h pH) similar to PSE-like meat. There may be an underlying effect of dietary phytase inclusion on final pH. Phytase may have provided inorganic phosphate ions as a means to produce energy during the initial stages of anaerobic metabolism, and/or modulated the efflux of lactate and protons from muscle through the MCT1 gene [66,67]. A more thorough investigation of phytase regulation in muscle metabolism may be favorable to better understanding the phytase relationship with pH.

Contrariwise to the effects of pH and drip loss to produce PSE-like meat, relative lightness (L*) was darker for heat-stressed broilers than that observed in the thermoneutral group, which is in agreement with a previous study [68]. This could be associated with enhanced mitochondrial activity and maintenance of dark deoxymyoglobin under HS conditions [69,70]. Furthermore, the upregulation of HSPs, at least HSP70, could contribute to this coloration as a strong association between HSP expression and meat color was recently postulated in porcine and bovine muscles [71,72]. Additionally, the oxidation status of myoglobin is known as a predominant factor in affecting meat color [73], and the dysregulated expression of GPX1 in our experimental condition could explain, at least partly, the effect of HS on breast color [74,75], however further in-depth investigations are needed. It is also possible, as it is involved in many signaling pathways and in meat tenderness [76,77], that Col1A1/2 dysregulation may contribute to broiler meat color under HS conditions.

A dietary effect was noted at both 4- and 24-h postmortem for instrumental b* values. The NC diet expressed more yellowness in breast fillets than both the PC and QB treatments. Currently, the effects of phytase on meat color have not been well defined and the availability of resources assessing the effects of inclusion on meat quality has not been reported. However, it is interesting to note that when phytase is absent, and P and Ca are deficient from the diet (NC), an increase in observed yellowness was noted. The need to better understand the correlation between phytase inclusion and “the bluing” effect that it may have on breast meat should be further assessed. In addition, HS reduced the yellowness in fillets when compared to the TN group. A net reduction of 1 point was observed in b* values between the two environmental groups. In the experiment by Zhang and colleagues [62], no differences were observed in b* values in breast meat between HS and TN groups. However, differences were observed in thigh meat b* values. The opposite trend was noted in the current experiment in breast b* values, and this would follow the same vein of explanation as for the meat darkness and the associated genes described above.

## 5. Conclusions

In summary, the known adverse effects of HS were present through meat quality analysis in the current experiment. Although resting pH and the limited drip loss aligned with PSE-like meat, the authors cannot confirm the presence of PSE-like meat in the current experiment. While QB supplementation enhanced growth performances and reduced the severity of muscle myopathies, limited improvements were noted in broiler meat quality. However, the interaction of phytase and environmental conditions on live performance may outweigh the lack of difference noted in meat quality. Future studies should assess the impact of titrated levels of phytase inclusion on broiler meat quality.

## Figures and Tables

**Table 1:** Composition of experimental ^1^ starter (d0–18), grower (d19–28), and finisher diets (d29–42) fed to Cobb 500 male broiler chicks containing adequate and suboptimal calcium and phosphorus levels.

	Starter		Grower		Finisher	
Item, % as-Fed	PC	NC	PC	NC	PC	NC
Corn	60.59	62.07	67.05	68.53	68.76	70.28
Soybean meal	33.39	33.19	26.27	26.07	24.46	24.23
Poultry fat	1.58	1.06	2.46	1.94	2.87	2.35
Dicalcium phosphate	1.99	1.18	1.89	1.08	1.69	0.88
Limestone	0.87	0.92	0.83	0.88	0.76	0.80
Salt	0.30	0.29	0.29	0.29	0.29	0.29
Sodium bicarbonate	0.27	0.27	0.28	0.28	0.28	0.28
Premix ^2^	0.22	0.22	0.22	0.22	0.22	0.22
DL-Methionine	0.30	0.30	0.26	0.26	0.24	0.23
L-Lysine-HCl	0.25	0.26	0.24	0.24	0.23	0.23
L-Threonine	0.11	0.11	0.06	0.05	0.05	0.05
L-Valine	0.09	0.08	0.08	0.07	0.06	0.06
Choline chloride, 60%	0.05	0.04	0.08	0.08	0.09	0.09
Calculated composition, % unless noted otherwise						
Ca	0.90	0.74	0.84	0.68	0.76	0.60
Available P	0.45	0.30	0.42	0.27	0.38	0.23
Na	0.20	0.20	0.20	0.20	0.20	0.20
AME, kcal/kg	2975	2975	3100	3100	3150	3150
CP	21.2	21.3	18.4	18.5	17.7	17.7
Digestible lysine	1.20	1.20	1.02	1.02	0.97	0.97
Digestible methionine	0.61	0.61	0.54	0.54	0.51	0.51
Digestible TSAA	0.90	0.90	0.80	0.80	0.76	0.76
Digestible threonine	0.82	0.82	0.66	0.66	0.63	0.63

^1^ Broilers received one of three dietary treatments containing adequate calcium and phosphorus levels (Positive control; PC), suboptimal calcium and phosphorus levels (Negative control; NC), and NC + 2000 FTU phytase added on top. ^2^ Premix supplied (per kg of complete feed): calcium, 171 mg; manganese, 120 mg; zinc, 120 mg; iron, 18 mg; copper, 18 mg; iodine, 1.44 mg; selenium, 0.3 mg; dl-α-tocopherol, 198 mg; niacin, 154 mg; d-pantothenie acid, 40 mg; riboflavin, 26 mg; pyridoxine, 11 mg; retinol, 9 mg; thiamine, 6 mg; menadione, 6 mg; folic acid, 4 mg; cholecalciferol, 0.55 mg; biotin, 0.33 mg; cobalamin, 0.05 mg.

**Table 2:** Effects of phytase supplementation on growth performance of heat-stressed broilers.

Treatments ^1^	Thermoneutral (TN)			Heat Stress (HS)				*p* Value		
Parameters ^2^	NC	PC	NC + QB	NC	PC	NC + QB	SEM	E	T	ExT
**d0–42**										
BW (g)	2231.9	2463.8	2465.1	2205.7	2349.1	2363.5	35.5	0.005	<0.001	0.40
FCR	1.640	1.711	1.662	1.524	1.651	1.641	0.070	0.10	0.12	0.62
WCR	3.714	3.711	3.735	4.131	4.457	4.452	0.190	<0.0001	0.26	0.29
	**TN**			**HS**						
WCR3	3.721 ± 0.081 ^a^			4.347 ± 0.073 ^b^				<0.0001		
BW (g)	2386.933 ± 25.2 ^a^			2306.1 ± 31.4 ^b^				0.04		
				**PC**						
	**NC**			**NC + QB**						
BW (g)				2406.45 ± 24.3 ^b^				<0.0001		
	2218.8 ± 21.1 ^a^			2414.3 ± 27.2 ^b^						
**d0–28**										
BWG d0–28	1525.9	1553.6	1564.5	1548.4	1533.7	1562.4	43.65	0.99	0.82	0.89
FCR	1.416	1.465	1.463	1.395	1.479	1.453	0.042	0.87	0.27	0.91
WCR	3.480	3.287	3.220	3.436	3.271	3.230	0.092	0.79	0.06	0.94
**d29–42**										
BWG d29–42	1335.6	1420.4	1513.7	1477.2	1440.0	1354.4	61.56	0.99	0.89	0.07
FCR	1.756	1.687	1.569	1.435	1.498	1.544	0.088	0.02	0.89	0.27
FCR ^3^	1.671 ± 0.060 ^a^			1.492 ± 0.047 ^b^				0.03		
WCR	3.682	3.565	3.621	4.286	4.834	5.005	0.194	<0.0001	0.25	0.12
WCR ^3^	3.623 ± 0.120 ^a^			4.708 ± 0.137 ^b^				<0.0001		

^a,b^ Means without a common superscript were determined to be significantly different (*p* ≤ 0.05) by a Tukey’s HSD test. ^1^ Experimental diets used. NC—Negative Control: deficient in available Ca and P; PC—Positive Control: standard diet; QB—Quantum Blue: NC diet supplemented by 2000 phytase units (FTU)/kg. ^2^ BW, body weight; BWG, body weight gain; FCR, feed conversion ratio; WCR, water conversion ratio.^3^ When main effect of environment was significant without interaction with treatment, results were pooled across environment and compared. Different superscript letters indicate a significant difference at *p* < 0.05.

**Table 3:** Effect of phytase supplementation on blood gas, electrolytes, glucose, hematocrit, and hemoglobin concentrations in broilers exposed to one of two environmental temperatures processed at 42 d of age.

Treatments ^1^	Thermoneutral (TN)			Heat Stress (HS)			*p* Value		
Parameters ^2^	NC	PC	NC + QB	NC	PC	NC + QB	E	T	ExT
pH	7.37 ± 0.03	7.31 ± 0.01	7.37 ± 0.02	7.39 ± 0.03	7.37 ± 0.01	7.42 ± 0.01	0.03	0.06	0.78
pCO_2_ (mm Hg)	44.42 ± 2.17	54.17 ± 1.31	49.66 ± 3.00	45.47 ± 3.54	47.17 ± 2.86	40.71 ± 1.92	0.02	0.06	0.13
PO_2_ (mm Hg)	41.25 ± 1.97	36.50 ± 1.92	39.12 ± 1.36	41.85 ± 1.09	38.37 ± 1.34	39.50 ± 2.07	0.48	0.06	0.89
HCO_3_ (mmol/L)	27.56 ± 0.63	27.70 ± 0.85	28.96 ± 0.84	27.54 ± 1.09	27.27 ± 0.97	26.60 ± 1.03	0.21	0.94	0.40
BE (mmol/L)	2.12 ± 0.73	1.42 ± 1.04	3.75 ± 0.86	2.71 ± 1.42	2.12 ± 0.97	2.12 ± 1.00	0.89	0.53	0.45
SO_2_ (%)	73.62 ± 2.56	61.42 ± 3.74	70.50 ± 3.11	76.28 ± 2.07	70.25 ± 2.86	74.12 ± 3.0	0.04	0.01	0.53
TCO_2_ (mmol/dL)	29.12 ± 0.69	29.28 ± 0.91	30.62 ± 0.88	29.00 ± 1.16	28.75 ± 1.07	27.75 ± 1.02	0.14	0.98	0.31
Na (mmol/L)	146.37 ± 1.23	147.62 ± 2.47	146.75 ± 0.76	144.00 ± 0.83	147.62 ± 1.27	144.87 ± 0.41	0.19	0.17	0.64
K (mmol/L)	5.65 ± 0.21	5.81 ± 0.15	5.47 ± 0.11	5.55 ± 0.23	5.61 ± 0.10	5.33 ± 0.10	0.29	0.18	0.94
iCa (mmol/L)	1.50 ± 0.01	1.42 ± 0.02	1.39 ± 0.02	1.37 ± 0.02	1.31 ± 0.05	1.35 ± 0.02	<0.01	0.05	0.30
Glucose (mg/dL)	253.25 ± 5.95	234.50 ± 4.4	234.50 ± 5.8	222.57 ± 4.40	227.25 ± 8.26	226.25 ± 4.5	<0.01	0.34	0.07
Hematocrit (%)	21.5 ± 0.95	22.87 ± 1.28	21.50 ± 0.5	21.57 ± 0.69	21.00 ± 0.82	21.75 ± 0.70	0.46	0.88	0.40
Hb (g/dL)	7.31 ± 0.31	7.77 ± 0.43	7.30 ± 0.16	7.34 ± 0.23	7.13 ± 0.16	7.38 ± 0.24	0.47	0.89	0.39
	**TN**			**HS**					
pH ^3^	7.35 ± 0.02 ^a^			7.40 ± 0.01 ^b^			0.05		
pCO_2_^3^	44.41 ± 1.71 ^a^			49.43 ± 1.59 ^b^			0.04		
sO_2_ ^3^	68.83 ± 2.09			73.44 ± 1.67			0.09		
iCa ^3^	1.44 ± 0.02 ^a^			1.35 ± 0.02 ^b^			<0.01		
Glucose ^3^	240.25 ± 3.67 ^a^			225.48 ± 3.58 ^b^			<0.01		
	**NC**		**PC**		**QB**				
sO_2_ ^4^	74.87 ± 1.71 ^a^		66.13 ± 2.58 ^b^		72.31 ± 2.23 ^a^			0.02	
iCa ^4^	1.44 ± 0.02		1.37 ± 0.03		1.37 ± 0.02			0.07	

^a,b^ Means without a common superscript were determined to be significantly different (*p* ≤ 0.05) by a Tukey’s HSD test. ^1^ Experimental diets used. NC—Negative Control: deficient in available Ca and P; PC—Positive Control: standard diet; QB—Quantum Blue: NC diet supplemented by 2000 phytase units (FTU)/kg. ^2^ pCO_2_, carbon dioxide partial pressure; pO_2_, partial pressure of oxygen; HCO_3_, bicarbonate; BE, base excess of the extracellular fluid; SO_2_, oxygen saturation; TCO_2_, total carbon dioxide; Na, sodium; K, potassium; iCa, ionized calcium; Hb, hemoglobin. ^3^ When the main effect of the environment was significant without interaction with treatment, results were independently compared across environments. ^4^ When the main effect of treatment was significant without interaction with the environment, results were independently compared across treatments.

**Table 4:** Meat quality characteristics of broilers fed one of three experimental diets and exposed to one of two environmental temperatures processed at 42 d of age.

	Thermoneutral (TN) ^1^			Heat Stress (HS) ^1^			*p*-Value			
Parameters	NC ^2^	PC ^2^	QB ^2^	NC ^2^	PC ^2^	QB ^2^	SEM	Diet	Temp	Diet X Temp
Drip Loss (%) ^3^	1.16	1.16	1.11	0.44	0.30	0.44	0.084	0.695	<0.0001	0.473
pH ^4^										
4 h PM	5.95 ^ab^	5.98 ^a^	5.91 ^b^	5.97 ^a^	5.97 ^a^	5.99 ^a^	0.014	0.113	0.009	0.010
24 h PM	5.81 ^b^	5.94 ^a^	5.84 ^b^	5.72 ^c^	5.70 ^c^	5.70 ^c^	0.022	0.078	<0.0001	0.040
Color ^5^										
4 h L* PM	52.96	53.39	53.79	52.34	52.01	52.83	0.318	0.284	0.015	0.698
24 h L* PM	53.41	53.64	53.89	51.24	51.48	51.77	0.306	0.399	<0.0001	0.998
4 h a* PM	3.80	3.77	4.03	3.98	3.98	4.11	0.124	0.193	0.112	0.846
24 h a* PM	3.74	3.44	3.77	3.44	3.66	3.52	0.130	0.883	0.461	0.315
4 h b* PM	9.05	8.84	8.49	8.83	7.57	7.91	0.202	0.012	0.020	0.404
24 h b* PM	8.95	9.04	8.05	7.96	7.09	7.04	0.175	0.001	<0.0001	0.995
		**TN**			**HS**					
Drip Loss (%) ^6^	1.14 ± 0.05 ^a^			0.39 ± 0.04 ^b^					<0.0001	
Color										
4 h L* PM ^6^	53.38 ± 0.26 ^a^			52.39 ± 0.23 ^b^					0.008	
24 h L* PM ^6^	53.65 ± 0.17 ^a^			51.49 ± 0.21 ^b^					<0.0001	
4 h b* PM ^6^	8.69 ± 0.16 ^a^			8.10 ± 0.20 ^b^					0.031	
24 h b* PM ^6^	8.35 ± 0.17 ^a^			7.36 ± 0.16 ^b^					0.0004	
	**NC**		**PC**		**QB**					
Color										
4 h b* PM ^7^	8.93 ± 0.26 ^a^		8.05 ± 0.27 ^b^		8.20 ± 0.14 ^ab^			0.014		
24 h b* PM ^7^	8.45 ± 0.26 ^a^		7.57 ± 0.19 ^b^		7.54 ± 0.21 ^b^			0.013		

^a–c^ Means without a common superscript were determined to be significantly different (*p* < 0.05) by a Tukey’s HSD test. ^1^ Environmental temperature conditions. Thermoneutral was maintained as a stepwise reduction to match breeder recommendations and heat stress utilized an 8-h heat phase 36 °C (8:30 a.m.–4:30 p.m.) from day 29 to d42. ^2^ Experimental diets used. NC—Negative Control: deficient in available Ca and P; PC—Positive Control: standard diet; QB—Quantum Blue: NC diet supplemented by 2000 phytase units (FTU)/kg. ^3^ Drip loss was calculated as percent (%) loss at 24-h postmortem in relation to initial deboned fillet weight. ^4^ pH was assessed using a spear tip probe inserted near the wing joint attachment of butterfly fillets at 4- and 24-h PM (postmortem). ^5^ Color was assessed on the dorsal side of deboned breast butterflies at 4- and 24-h PM (postmortem) using a calibrated colorimeter to determine L* (relative lightness; 0–100), a* (relative redness; −60 to +60), and b* (relative yellowness; −60 to +60) values using a D65 parameter. ^6^ When the main effect of the environment was significant without interaction with the treatment, results were independently compared across environments. ^7^ When the main effect of treatment was significant without interaction with the environment, results were independently compared across diets.

## Data Availability

Data sharing not applicable.
